# Sacroplasty with or without screw fixation for sacral metastatic tumors

**DOI:** 10.3389/fonc.2025.1494377

**Published:** 2025-02-26

**Authors:** Evan P. Cox, Sean Tutton, Matthew Scheidt, Brandon M. Key, John C. Neilson, Adam N. Wooldridge, Meena Bedi, Donald A. Hackbarth, David M. King

**Affiliations:** ^1^ Department of Orthopaedic Surgery, Medical College of Wisconsin, Milwaukee, WI, United States; ^2^ Department of Vascular and Interventional Radiology, Medical College of Wisconsin, Milwaukee, WI, United States; ^3^ Department of Radiation Oncology, Medical College of Wisconsin, Milwaukee, WI, United States

**Keywords:** percutaneous screw fixation, sacroplasty, sacral metastases, pathological fracture, impending fracture, cementation, screw augmentation

## Abstract

**Introduction:**

Cementation (sacroplasty) with or without ablation has been shown to improve pain and function for patients with sacral metastatic disease. Percutaneous screw fixation with sacroplasty (PSFS) may provide superior outcomes in select patients.

**Methods:**

Thirty patients with sacral metastases who underwent sacroplasty with or without ablation and screw fixation at a single institution were retrospectively reviewed. Patients were compared based on treatment (PSFS or sacroplasty alone) and fracture status (pathological or impending) with an ANCOVA. Traumatic fractures were excluded. Patients were followed for 4.4 months on average (range, 2 weeks to 36.5 months). Functional outcomes were assessed using the Musculoskeletal Tumor Society (MSTS) score. The rate of secondary procedures as well as changes in narcotic usage were noted.

**Results:**

Patients with pathological fractures who underwent PSFS demonstrated increased postoperative MSTS scores compared to those who underwent sacroplasty (51% ± 19 versus 25% ± 13, p = 0.005). Patients with impending pathological fractures who underwent PSFS did not demonstrate statistically significant increased postoperative MSTS scores compared to those who underwent sacroplasty alone (38% ± 17 versus 32% ± 12, p = 0.72).

**Discussion:**

PSFS may provide additional benefit for patients with pathological fractures, while sacroplasty alone may be sufficient for those with impending pathologic fractures secondary to sacral metastatic disease. This study was limited by its retrospective design and sample size; however, the results may aid in treatment indications for sacral metastases and guide further research *Level of Evidence* Level III, Therapeutic Study.

## Introduction

**Figure 1:**
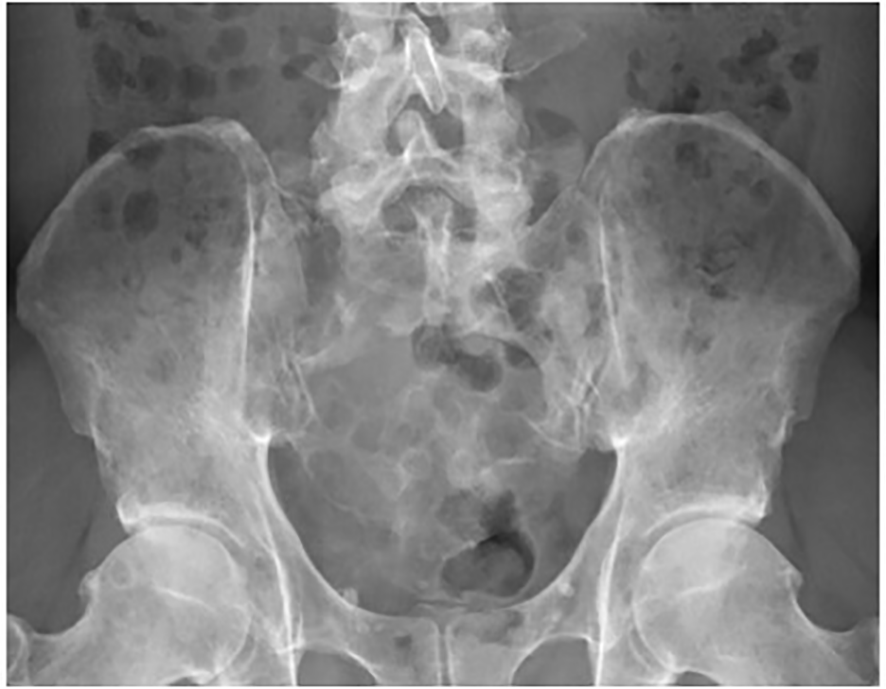
A 46-year-old male with extensive destruction of the sacrum and bilateral iliac bones secondary to multiple myeloma. Preoperative radiograph.

Metastatic sacral tumors are a rare occurrence in cancer patients. Roughly 10% of cancer patients will have symptomatic spinal metastases with the minority of cases involving the sacrum (1, 2). Patients with sacral metastases will often experience disabling pain and mechanical instability due to pathological fractures or impending pathological fractures (**Figure 1**). The persistent pain associated with metastatic bone disease is often debilitating and may subject patients to chronic opioid use. Treatment options focus primarily on pain palliation and improving the quality of life for these patients by reducing the risk of neurological complications. Clinical interventions have typically involved open surgery, radiation therapy (RT), chemotherapy, and/or analgesic therapy (3). Due to the potential high morbidity risks of open surgery, patients with advanced metastatic disease are not good surgical candidates.

Minimally invasive procedures including cementation (sacroplasty) with or without thermal ablation have been shown to improve pain and provide bone stabilization, with minimal risk to the patient (4–7). Although effective, there are still patients who do not experience sufficient pain resolution due to extensive bone destruction. Percutaneous screw fixation with sacroplasty (PSFS) may increase mechanical stability and improve outcomes in select patients. The treatment of sacral metastatic disease is complex, and there are no standard criteria for treatment at the present time.

The purpose of this study was to determine if PSFS can provide additional benefits for patients with painful metastatic disease to the sacrum. Additionally, we would like to determine when sacroplasty without additional screw fixation may be sufficient.

## Materials and methods

After obtaining institutional review board approval, we performed a retrospective review of all patients with sacral metastatic disease who underwent PSFS or sacroplasty alone at our institution from 2012 to 2022. Inclusion criteria included impending or minimally displaced pathological fractures secondary to sacral metastatic disease. Impending fractures were defined as areas of symptomatic osteolytic metastasis without a clear cortical fracture line observed on CT scan. High-energy traumatic sacral fractures were excluded from this study. Thirty patients met the inclusion criteria for this study. Demographic data pertaining to the cohort of this study is summarized in **Table 1**. Twenty-one patients were found to have sustained pathological fractures, while 9 patients had impending pathological fractures. Of the 21 patients with pathological fractures, 9 underwent PSFS, while 12 underwent sacroplasty alone. Of the 9 patients with impending pathological fractures, 2 underwent PSFS, while 7 underwent sacroplasty alone. The patients were followed for 4.4 months on average (range, 2 weeks to 36.5 months). Initial follow-up visit was at 1-2 weeks, with additional visits requested at 1 month and three months thereafter. Often these visits coincided with medical oncology visits for patient convenience. If the patient felt symptom resolution after the initial follow-up visit, an additional visit was often not required.

**Table 1:** Demographic data.

Characteristic	PSFS Patients (N = 11)	Sacroplasty Patients (N = 19)
Mean age (yr) Sex Male Female Cancer subtype (no. of patients) Multiple myeloma Liver, Renal Other Prior local radiation therapy (no. of patients) Ablation (no. of patients) Overall Radiofrequency ablation Cryoablation Microwave ablation	63 8 (73%) 3 (27%) 5 (46%) 4 (36%) (2 each) 2 (18%) 5 (46%) 3 (27%) 1 (33%) 1 (33%) 1 (33%)	65 8 (42%) 11 (58%) 8 (42%) 2 (11%) (1 each) 9 (47%) 8 (42%) 7 (37%) 6 (86%) 1 (14%) 0 (0%)

Functional outcomes were assessed with the use of the 1993 Musculoskeletal Tumor Society (MSTS) score (1). These scores reflected data from the patients’ preoperative and final postoperative clinical encounter with either the orthopedic surgery team (D.M.K., J.C.N., A.W., D.H.) or interventional radiology team (S.M.T., M.S., B.K.). Postoperative changes in narcotic usage were gleaned from the medical record. The use of narcotics was not quantified. Potential confounding variables from our study cohort were prior local RT and thermal ablation technique used.

Descriptive statistics were calculated. To control for the potential covariate preoperative MSTS scores, an analysis of covariance (ANCOVA) was used to identify a significant difference in the postoperative increase in mean total MSTS scores between patients undergoing PSFS and sacroplasty alone for pathological fractures and impending pathological fractures. Level of significance was set at p <0.05

To address the potential confounding variable of prior local RT, we evaluated the RT frequency between both groups (PSPF and sacroplasty alone). The frequency of prior local RT in PSFS and sacroplasty alone patients was determined to be 46% and 42%, respectively. With our study design, the similar frequencies led us to believe that prior local RT would not negatively impact the results of this study.

### Procedural technique

Surgical indications for PSFS included several factors: (1) Large destructive lesions showing extensive osseous involvement. (2) Persistent pain despite undergoing RT. (3) Mechanical pain limiting ambulation in need of stabilization. Ablation was performed prior to PSFS or sacroplasty for patients with radioresistant tumors such has renal cell lesions. Malignant lesions that respond well to radiation (breast, lymphoma or myeloma) were typically not ablated. Careful consideration was taken into account when deciding on which method of ablation would be optimal. Cryoblation was typically used on larger tumors with multiple adjacent treatment zones and tumors with a soft-tissue component that was within close proximity of neurovascular structures or articular cartilage. Radiofrequency or microwave ablation was chosen for smaller intraosseous tumors.

**Figure 2:**
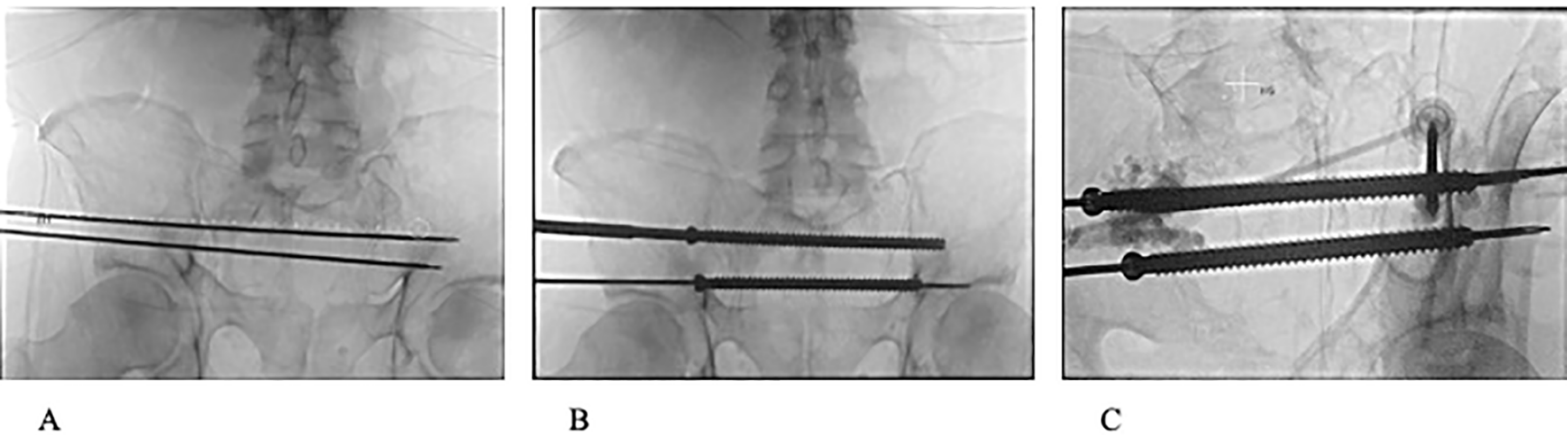
**(A)** Fluoroscopic view of 2 3.2mm guide pins passing through S1 and S2 corridors. **(B)** Fluoroscopic view of 8 mm cannulated screws passed through S1 and S2 corridors. **(C)** Fluoroscopic image during PMMA injection with 2 11-guage cannulas into weakened portions of the ilium and reinforcing entrance and anchoring positions of S1 and S2 trans-sacral screws.

**Figure 3:**
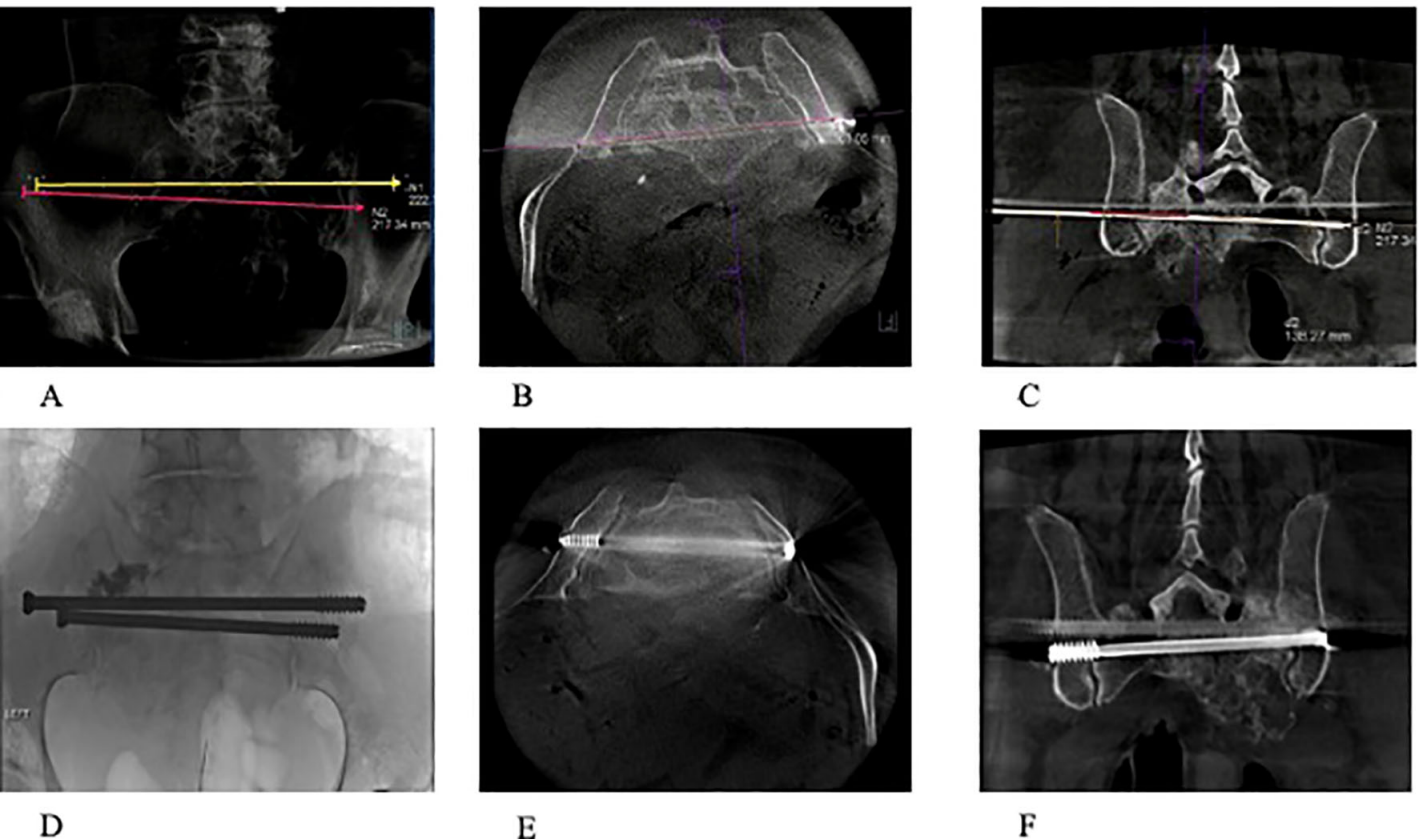
**(A–F)** A 74-year-old patient with osteoradionecrosis of pathological fracture secondary to metastatic pancreatic cancer. **(A–C)** Radiograph and CT scans (axial and coronal) showing the planned screw trajectories through S1 and S2 corridors. **(D–F)** Completion radiograph and CT scans (axial and coronal) demonstrating stabilization of the sacrum with the use of percutaneous trans-sacral screw fixation and sacroplasty.

**Figure 4:**
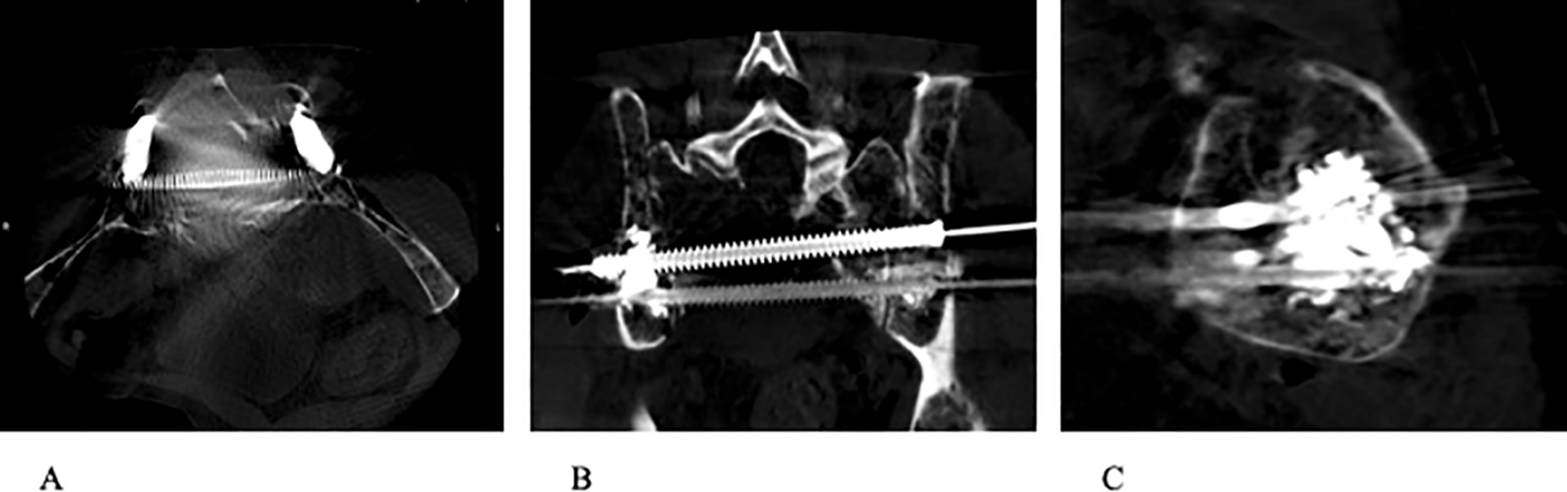
**(A–C)** Completion CT images (axial, coronal, and sagittal) demonstrating stabilization of sacrum with the use of percutaneous trans-sacral screw fixation and sacroplasty.

Initial CT images are obtained and then CT fluoro is used. Using angulation of the C-Arm, fluoroscopic images with augmented fluoroscopy needle guidance overlay were utilized to guide pins through sacral corridors. A cone beam CT was then performed to confirm appropriate placement of sacral guide pins without encroachment on the neural foramina. Fully threaded cannulated screws were then placed through S1 and S2 corridors. Either 8-mm or 6.5-mm (Stryker) or 7.3-mm (DePuy Synthes) cannulated screws were used (**Figure 2**). For each patient, the location, screw type (Ilio-sacral versus trans-sacral), and number of screws was decided based on achieving optimal fixation for improved stabilization, allowing for improved functional outcomes (**Figure 3**). Using fluoroscopic needle guidance, 11-gauge cannulas are either placed using the posterior to anterior approach, or trans-sacroiliac approach. Under CT fluoroscopic guidance, Polymethylmethacrylate (PMMA) cement was then injected under CT fluoroscopic guidance filling the lesion and augmenting screw fixation (**Figure 2**). Real-time fluoroscopy with overlaying 3-dimensional CT imaging provides superior visualization of the neuroforamina, which ensures good cement deposition and with minimal extravasation. When necessary, additional trocars were used to maximize cement filling of osseous defects and ensure screws were “potted” into the cement. Final CT images were taken prior to closure (**Figure 4**).

**Figure 5:**
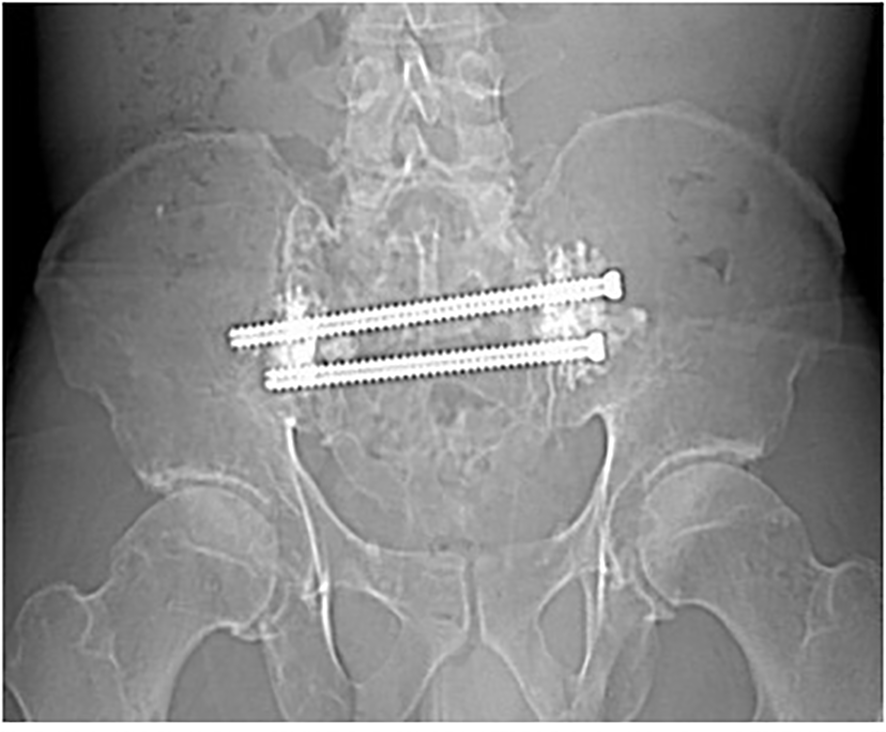
Radiograph made 9 months following CT-Fluoroscopic guided trans-sacral screw fixation with sacroplasty, showing stability with no evidence of screw loosening.

Incisions were closed with 3-0 Monocryl sutures and Dermabond. Following the procedure, a 1-week follow-up visit was standard, with additional visits being requested at 1 month and 3 months thereafter, while also attempting to schedule visits that coincide with other medical oncology visits the patient may have. Radiographs were obtained during follow up visits (**Figure 5**).

## Results

Patients with pathological fractures who underwent PSFS demonstrated a much greater increase in MSTS scores postoperatively compared to those who underwent sacroplasty alone (51% ± 19 versus 25% ± 13, p = 0.005). The mean MSTS score for PSFS patients increased from 7.3 to 22.6 of 30 postoperatively, compared to sacroplasty patients which increased from 10.7 to 18.14 of 30 postoperatively (**Table 2**). The 9 patients who underwent PSFS demonstrated greater improvements in all aspects of the MSTS score and did not require secondary procedures due to unresolved pain following the procedure. Six (67%) of the 9 PSFS patients reported a decrease in narcotic usage, and the remaining 3 (33%) patients reported stable usage following the procedure. Four (33%) of the 12 sacroplasty patients reported a decrease in narcotic usage, 3 (25%) patients reported increased narcotic usage, and the remaining 5 (42%) patients reported stable usage. The secondary procedure rate for PSFS and sacroplasty patients was 0% and 42%, respectively. Secondary procedures were required at a mean of 197 days, median 90 days (range 14 to 730 days) and consisted of minimally invasive interventions (sacroiliac joint steroidal injections, RT, neurolysis, or additional sacroplasty). The differences seen in postoperative increases in MSTS scores in combination with secondary procedures rates demonstrate that PSFS may offer additional beneficial for patients with pathological fractures secondary to sacral metastatic disease.

**Table 2:** MSTS scores for patients with pathological fractures.

Table II MSTS Scores*					
Pathological Fractures	PSFS Patients (N = 9)			Sacroplasty Patients (N = 12)	
	Preoperative	Postoperative	P Value	Preoperative	Postoperative
Total Pain Emotional Acceptance Use of supports Walking ability Gait Function	7.3 0.2 0.4 0.9 1.6 2.8 1.3	22.6 3.8 4.2 3.0 3.6 4.4 3.6	0.005 0.021 0.004 0.014 >0.05 0.021 >0.05	10.7 0.9 1.0 1.8 2.2 3.0 1.8	18.1 2.8 2.8 2.3 3.1 3.8 3.3

*Musculoskeletal Tumor Society Scoring system; a score of 0 to 5 is assigned in each of 6 categories, which are then combined for a maximum possible score of 30.

Patients with impending pathological fractures who underwent PSFS demonstrated a greater increase in MSTS scores postoperatively compared to those who underwent sacroplasty alone, but the difference was not statistically significant (38% ± 17 versus 32% ± 12, p = 0.72). The mean MSTS score for PSFS patients increased from 12 to 23.5 of 30, compared to sacroplasty patients which increased from 18.4 to 28 of 30 (**Table 3**). Five (71%) of the 7 sacroplasty patients reported a decrease in narcotic use, and the remaining 2 (29%) reported stable usage following the procedure. One (50%) of the 2 PSFS patients reported a decrease in narcotic usage, while the other patient reported an increase in narcotic usage following the procedure. The secondary procedure rate for PSFS and sacroplasty alone patients was 0% and 14%, respectively. One patient treated with sacroplasty alone underwent sacroiliac joint steroidal injection 7 months postoperatively due to ongoing pain. With a low secondary procedure rate in addition to similar increases observed in postoperative MSTS scores, sacroplasty without additional fixation may be sufficient for patients with impending pathological fractures secondary to sacral metastatic disease.

**Table 3:** MSTS scores for patients with impending pathological fractures.

Table III MSTS Scores*					
Impending Fractures	PSFS Patients (N = 2)			Sacroplasty Patients (N = 7)	
	Preoperative	Postoperative	P Value	Preoperative	Postoperative
Total Pain Emotional Acceptance Use of supports Walking ability Gait Function	12 0.5 0.5 4.0 3.0 3.0 1.0	23.5 3.0 3.5 5.0 4.5 4.0 3.5	>0.05 >0.05 >0.05 >0.05 >0.05 >0.05 >0.05	18.4 1.7 1.4 4.4 3.7 4.1 3.0	28.0 4.9 4.9 5.0 4.7 4.7 4.4

*Musculoskeletal Tumor Society Scoring system; a score of 0 to 5 is assigned in each of 6 categories, which are then combined for a maximum possible score of 30.

One procedural complication was observed with one patient with a pathological fracture secondary to multiple myeloma. This patient underwent S1 and S2 trans-sacral screw fixation for a large pathological fracture to the right sacral sala. The patient experienced right buttock discomfort 5 months postoperatively, and imaging demonstrated the S1 screw had backed out. The S1 screw was subsequently removed successfully with no further complications observed and the patient experienced improved function and pain relief.

## Discussion

Open sacral surgery and minimally invasive interventions such as radiation therapy and sacroplasty have been the treatment options for pathological fractures due to sacral metastatic disease. However, open surgery carries a morbidity risk and patients may still experience ongoing pain following radiation therapy and/or sacroplasty. The treatment of sacral metastatic disease remains a challenge, and a standard criterion for treatment has yet to be established.

Papanastossiou et al. (8) reported on a series of 6 oncologic patients with sacral insufficiency fractures who underwent percutaneous SI screw fixation under fluoroscopic guidance. Previous studies reporting on navigated SI screw fixation have focused primarily on the use of 1 screw per level. The authors proposed a modification to this technique in which multiple long screws may be inserted per level to achieve optimal fixation. Fluoroscopic image guidance was found to be helpful in increasing the accuracy of screw placement. One revision was required for an S2 screw for one patient who experienced S1 radiculopathy. Aside from this revision, all patients experienced favorable outcomes postoperatively. Although flouroscopic-guided SI fixation was found to be safe and effective, it carries a screw malposition incidence rate from 2% up to 15% (9).

Trumm et al. (10) described the PSFS technique using CT and fluoroscopic guidance which allows for precise targeting resulting in fracture stabilization and minimized risk for neuroforaminal extravasation. It remains unclear which subset of patients will experience an additional benefit from this treatment as opposed to undergoing conventional sacroplasty. The current study demonstrates that PSFS may offer additional benefits for patients with pathological fractures secondary to sacral metastatic disease. In comparison to those treated with sacroplasty alone, PSFS lead to superior outcomes regarding overall functioning, decreased narcotic usage, and secondary procedure rates. For patients with impending pathological fractures however, sacroplasty without additional screw fixation may be sufficient.

The rebar and cement concept used in PSFS reduces the risk of screw dislodgment and improves mechanical stability, with limited risk to the patient. PMMA cement deposition provides optimal resistance against compressive forces across large lytic defects. Cementation further stabilizes the screws within areas of osseous destruction, which helps minimize screw motion that can lead to poor healing. Maximum interdigitation between hardware and cement is likely achieved with the use of fully threaded screws instead of partially threaded screws. Together, this combination enhances the rotational and torsional stability of the sacrum. Several reports have shown this method to be safe and effective in treating sacral pathological fractures as well (10–15). In a recent case report, Galmich et al. (16) discusses a patient with a pelvic radiation induced pathological fracture of the sacrum who was initially treated with sacroplasty alone. Sacroplasty was not found to be sufficient in providing pain resolution one month later. A decision was made to treat the patient with PSFS under CT-Fluoroscopic guidance. The patient tolerated the procedure well with no complications. Immediate pain relief was observed following the procedure. At 6 months follow up, the patient’s condition remained stable. They concluded that sacroplasty alone may not be sufficient for symptom relief and fracture stabilization for large bone defects or complex fractures. Despite its efficacy being demonstrated in recent studies, literature that compares this approach to sacroplasty in the setting of sacral metastatic disease has been limited. To our knowledge, this is the first study that compares the two treatment approaches, and assesses when PSFS may be the preferred option for treating pathological fractures secondary to sacral metastatic disease.

Dehdashti et al. (17) first described the use of sacroplasty as a palliative treatment for sacral metastatic lesions. Biomechanical stability and immediate symptom relief is achieved with percutaneous PMMA injection into the focal lesions of the sacrum. Sacroplasty has now been well established in the literature as an effective palliative treatment for patients with sacral metastatic disease (2, 6, 18). The results of the current study regarding the safety and efficacy of sacroplasty are consistent with recent studies. Due to the varying degree of bone destruction seen with metastatic sacral tumors, our study assesses which subset of patients will sacroplasty be sufficient for. This was accomplished by reviewing the functional outcomes and the secondary procedure rate following sacroplasty. Due to advances in oncological treatments, cancer survivor has been increasing. The longevity of the beneficial effects with sacroplasty remains unclear, therefore it is important to address which patients would fare well with sacroplasty without the need for reinterventions. Our study provides data which demonstrates that sacroplasty without additional screw fixation may be sufficient for impending pathological fractures. Due to the 42% secondary procedure rate observed with patients with pathological fractures, it remains unclear if sacroplasty is sufficient for this subset of patients.

The current study is limited by its retrospective design and small sample size. Additionally, surgical indications were not clearly defined. The cohort was heterogeneous with respect to tumor subtype, fracture pattern, prior local RT, and thermal ablation technique used. Another limitation of the current study is the use of subjective measures such as MSTS scores and patient-reported narcotic usage. Although this study has its limitations, the conclusions drawn from this study may guide further research in the treatment of sacral metastatic disease.

## Conclusions

The treatment of metastatic sacral tumors continues to present difficult challenges. A standard criterion for treating sacral metastatic disease is still lacking today. Minimally invasive procedures such as sacroplasty, thermal ablation, and radiotherapy have been the mainstay of treatment. Although effective, there are still patients that may not experience complete pain resolution following these treatments. Our study provides data for when PSFS should be considered a primary option. Further research addressing the impact of fracture location, tumor subtype, and prior local radiotherapy may provide additional insight for when screws are more advantageous. Additionally, when treating patients with additional screw fixation, it remains unclear when screw placement should involve both S1 and S2. PSFS is a promising procedure which offers great benefit for this patient population; however, additional studies are necessary to aid in the establishment of criteria and guidelines for treating sacral metastatic disease.

## Data Availability

The original contributions presented in the study are included in the article/supplementary material. Further inquiries can be directed to the corresponding author.
